# Increasing Incidence of Canine Leptospirosis in Switzerland

**DOI:** 10.3390/ijerph110707242

**Published:** 2014-07-16

**Authors:** Andrea Major, Ariane Schweighauser, Thierry Francey

**Affiliations:** Department of Clinical Veterinary Medicine, Vetsuisse Faculty University of Bern, Länggassstrasse 128, CH-3001 Bern, Switzerland; E-Mails: andrea.major@vetsuisse.unibe.ch (A.M.); ariane.schweighauser@vetsuisse.unibe.ch (A.S.)

**Keywords:** leptospirosis, dog, zoonosis, climatic data, one health, renal failure, pulmonary hemorrhage

## Abstract

A marked increase in canine leptospirosis was observed in Switzerland over 10 years with a peak incidence of 28.1 diagnosed cases/100,000 dogs/year in the most affected canton. With 95% affected dogs living at altitudes <800 m, the disease presented a seasonal pattern associated with temperature (*r*^2^ 0.73) and rainfall (*r*^2^ 0.39), >90% cases being diagnosed between May and October. The increasing yearly incidence however was only weakly correlated with climatic data including number of summer (*r*^2^ 0.25) or rainy days (*r*^2^ 0.38). Serovars Australis and Bratislava showed the highest seropositivity rates with 70.5% and 69.1%, respectively. Main clinical manifestations included renal (99.6%), pulmonary (76.7%), hepatic (26.0%), and hemorrhagic syndromes (18.2%), leading to a high mortality rate (43.3%). Similar to the human disease, liver involvement had the strongest association with negative outcome (OR 16.3). Based on these data, canine leptospirosis presents similar features and severity as the human infection for which it therefore can be considered a model. Its re-emergence in a temperate country with very high incidence rates in canines should thus be viewed as a warning and emphasize the need for increased awareness in other species.

## 1. Introduction

Leptospirosis is an emerging infectious disease of global importance [1,2]. Caused by spirochetes of the pathogenic genus *Leptospira*, it is considered one of the most widespread zoonoses worldwide [2]. Different wildlife species including small rodents, as well as domestic livestock and dogs, may act both as maintenance and incidental hosts of a variety of serovars. Animals may be maintenance hosts of some serovars, like the dog is a host of serovar Canicola, or become sick as incidental hosts of other serovars [1,2]. Small mammals are the most relevant maintenance hosts and vectors of infection for livestock, domestic pets and humans by shedding the bacteria through their urine [2]. Infection may occur from exposure to urine of carrier animals either directly or via contamination of soil or water [1]. Important risk factors for zoonotic transmission of leptospires therefore include both environmental factors and human interactions with wildlife and companion animals [2]. Warm and humid conditions favor a longer survival of leptospires in the environment, resulting in a higher incidence of human leptospirosis in the tropics, and peak incidence in summer and fall in more temperate regions [1,2]. Similar findings are reported from veterinary medicine, where seasonal peaks of clinical disease correlate with local temperature and rainfall patterns [3]. Indirect transmission through water sources contaminated with infected urine is likely one of the major routes of infection of humans exposed during water-related recreational and occupational activities. Adventure travelers and sportsmen returning from tropical regions therefore account for at least part of cases of clinical disease in industrialized countries [4]. In central European countries, autochthonous leptospirosis has been reported with an incidence rate of 1.26 per 100,000 inhabitants per year in a study from South-East Austria [5,6]. The authors hypothesize further that a considerable number of clinical cases are not even reported due to diagnostic difficulties and lack of reporting compliance, and calculated incidences are likely underestimated. The most common reported risk factors for infection in this study included activities in the woods, wet areas, gardening, and exposure to rodents, while 18% of people had been in contact with dogs, 46% reported contact to mice and rats [5].

Several studies investigated the particular role of the dog as zoonotic vector. Seroprevalence of *Leptospira spp*. in seemingly healthy canines in different countries varied between 4.9% and 35.2% [7,8,9,10,11] and renal carriage or urinary shedding was documented by PCR in 1.5%–8.0% of dogs from various origins [12,13,14]. A large discrepancy between the fast rising number of diseased canines and the rare autochthonous human cases has recently been noted in Switzerland [15,16,17]. Even humans with intensive and frequent contact with dogs only rarely develop clinical infections with leptospires. Barmettler *et al*. investigated the risk of zoonotic transmission from dogs with acute leptospirosis to their owners and to the staff of a veterinary hospital with a high caseload of leptospirosis. Seroreactivity to *Leptospira* serovars in this human population was not detected in any of the 91 tested subjects and zoonotic transmission under standard recommended hygiene conditions was therefore deemed uncommon [18]. Interestingly, to the authors’ knowledge no proven case of direct dog-to-human transmission has been reported in the literature, although anecdotal cases are occasionally cited. Most of the evidence for the role of the dog as a relevant vector of infection is indirect and based on serological profile analogy [13,19].

On the other side however, dogs frequently exposed to water and those spending a lot of time outdoors such as herding and hunting dogs have been shown to be at increased risk of infection, similarly to humans [20,21]. This resemblance possibly argues more for a role of canines as victims of infection rather than culprits for its spread [19]. Furthermore, in view of the discrepant incidence of clinical disease between various species in some countries, we suggest that the dog may be an indicator of the presence of *Leptospira interrogans* in the environment [22].

The goals of this study include the description of leptospirosis as a re-emerging zoonotic disease in the canine population of Switzerland over a 10-year-period, and the evaluation of the effect of climatic factors on the changing incidence and the seasonality of the disease. We thereby wish to raise awareness for this potentially fatal zoonosis in the temperate areas.

## 2. Experimental Section 

### 2.1. Data Collection

The database of the Small Animal Teaching Hospital of the Vetsuisse Faculty of the University of Bern and the database of the Nephrology Group were searched for cases of dogs diagnosed with leptospirosis between January 2003 and December 2012. Signalment, presenting complaint, history, physical exam findings, laboratory results, and outcome data were extracted from the medical records. Only dogs with minimal datasets including complete medical records, hematology, biochemistry, and medical imaging were considered for inclusion.

Signalment data were further obtained from the general hospital canine population (hospital database) and from the Swiss national registry for dogs (ANIS database, Animal Identity Service AG, Bern, Switzerland) for the purpose of comparing signalment parameters and cantonal distribution of the normal canine population. 

Biogeographic data used for the characterization of the cases were obtained from the Swiss Federal Office for the Environment. For simplification, bioregions were grouped in Jura, Plateau, and Alps. Monthly and yearly climatic data, including temperature, rainfall and derived parameters (number of rainy days, winter days, freezing days, summer days) were obtained from the Swiss Federal Statistical Office for the corresponding time period.

### 2.2. Clinical and Laboratory Characterization

A case of leptospirosis was defined based on consistent clinical presentation, including dogs with renal, hepatic, pulmonary or hemorrhagic manifestations, with laboratory confirmation [18,23,24]. The latter could be obtained with either paired microscopic agglutination test (MAT) serology; single sample MAT serology; canine IgM ELISA; PCR on blood, urine, kidney or liver tissue; or consistent histopathology findings at necropsy [23]. 

Dogs with leptospirosis were classified clinically based on organ involvement using standard criteria defined for the canine species. Renal involvement or acute kidney injury (AKI) was defined as the combination of acute uremic syndrome, renal azotemia and urinary parameters indicating kidney injury [25]. Dogs with any evidence of underlying chronic kidney disease (CKD) were only considered renal if they had evidence of acute exacerbation of otherwise stable disease. Severity of AKI was defined based on the described IRIS grading system [26]: mild AKI (corresponding to IRIS grades 1–3; serum creatinine < 440 µmol/L), moderate AKI (corresponding to grade 4; serum creatinine 440–880 µmol/L), and severe AKI (corresponding to grade 5; serum creatinine > 880 µmol/L).

Hepatic involvement was defined as the presence of hepatic hyperbilirubinemia and arbitrarily considered relevant with serum bilirubin ≥ 10 µmol/L (normal: 0.5–4.0 µmol/L). It was further classified as mild for serum bilirubin 10–20 µmol/L and severe for bilirubin > 30 µmol/L [24,27]. Pulmonary involvement was defined as clinical evidence of relevant pulmonary disease causing labored breathing or dyspnea and/or radiographic evidence of moderate to severe peribronchial, interstitial or alveolar infiltrates [28,29,30,31]. Dogs with rapid resolution of pulmonary signs after correction of fluid overload, indicating iatrogenic pulmonary edema, were not considered to have the severe pulmonary form of leptospirosis (SPFL). Hemorrhagic syndrome (typically disseminated intravascular coagulopathy, DIC) was diagnosed in dogs with at least two abnormal parameters from the four routinely performed tests (platelet count, prothrombin time, activated partial thromboplastin time, and plasma fibrinogen concentration) [23]. 

Serology with MAT was performed by the accredited National Reference Laboratory for Leptospirosis (Institute of Veterinary Bacteriology, National Center for Zoonoses, Bacterial Animal Diseases and Antimicrobial Resistance, Vetsuisse Faculty, University of Bern, Switzerland) according to the Guidelines of the World Health Organization International Leptospirosis Society [32]. Briefly, the MAT was performed using a panel of ubiquitous and locally prevalent serovars, including *L. interrogans* serovars Australis, Autumnalis, Bataviae, Bratislava, Canicola, Hardjo, Icterohaemorrhagiae, Pomona, Sejroe, and Tarassovi and *L. kirschneri* serovar Grippotyphosa. Sera were initially screened at a dilution of 1:100. Samples with a positive reaction were titrated in a serial two-fold dilution to a maximum of 1:3200 and the end-point titer was recorded. For case definition, paired serology with fourfold rise in sequential titers at a 1–3 weeks interval was considered first-choice. For animals where a second sample could not be obtained (typically due to early death), a single sample obtained at presentation with a titer ≥1:800 was considered positive, as previously demonstrated [24]. No difference was made in the interpretation of vaccine and non-vaccine serovars since all vaccinated dogs positive for one of the 2 vaccine serovars Icterohemorrhagiae and Canicola also displayed a stronger positive reaction to at least another serovar. Since the results of MAT serology are considered poor predictors of the infecting serovar in humans, no attempt was made to define the infecting serovars and only rates of seropositivity and seroconversion are reported [33].

A point-of-care canine IgM ELISA (Test-it^®^ Canine Leptospira Lateral Flow Rapid Test, Life Assay Diagnostics, Cape Town, South Africa) was performed at presentation [34]. Because of possible false positive results following vaccination, the test was only used in dogs not vaccinated for leptospirosis in the 5 months prior to presentation.

The LipL32 nested PCR was performed by a commercial laboratory (IVD, Gesellschaft für Innovative Veterinärdiagnostik GmbH, Hannover, Germany) on blood, urine, kidney or liver tissue, depending on sample collection timing, tissue availability, and pre-referral treatment history. The proprietary genus-specific oligonucleotide primers included an external 519-bp product and an internal 286-bp product and were derived from the sequence of *L. interrogans* strain RZ11 (GenBank accession No. AF181553) [24].

### 2.3. Data and Statistical Analysis

In 2007, availability of renal replacement therapy for small animals at our hospital changed markedly the treatment of dogs affected with leptospirosis. At the same time, the diagnostic workup was standardized to include systematic screening for the four main organ manifestations in all affected dogs, whenever possible. Statistical analyses dealing with organ manifestations and outcome were therefore performed both for the whole study population (2003–2012) and for a restricted dataset from the 256 cases diagnosed between 2007 and 2012. Similarly, in order to avoid case selection and recruitment bias, analyses of geoclimatic data were performed on the whole dataset of 298 cases as well as in the 278 cases originating only from the 10 main cantons affected (defined as the cantons with more than five cases in 10 years), and in 90 cases originating only from the canton Bern where the university hospital is located. 

All data were retrieved from the hospital information system, stored and organized for statistical procedures in Microsoft Excel, and exported for statistical analysis to commercial statistical software (NCSS, version 8, NCSS LLC, Kaysville, UT, USA). Numerical data were tested for normality with the Shapiro-Wilk W test and the Kolmogorov-Smirnov test. Since multiple sets of data were not normally distributed, all numerical data are presented as median and interquartile range (IQR). Statistical comparisons between groups of numerical data were performed with the Mann-Whitney U test, where appropriate, and comparisons of proportions of categorical data were performed with the Fisher exact test or the Chi-square test. Correlations between climatic data and case numbers were tested with a single linear regression analyses and reported as the coefficient of determination *r*^2^ and the *P*-value. However, since the cases diagnosed at the authors’ institution were not necessarily representative for the whole Swiss canine population, associations with geoclimatic data are purely descriptive, aiming at identifying the main parameters associated with case numbers. 

Univariate odds ratios for negative outcome were calculated for the organ involvements with a logistic regression analysis and expressed with their 95% confidence intervals. For all statistical tests, *P*-values < 0.05 were considered significant.

## 3. Results and Discussion

### 3.1. Results 

#### 3.1.1. Dogs: Signalment

**Figure 1:**
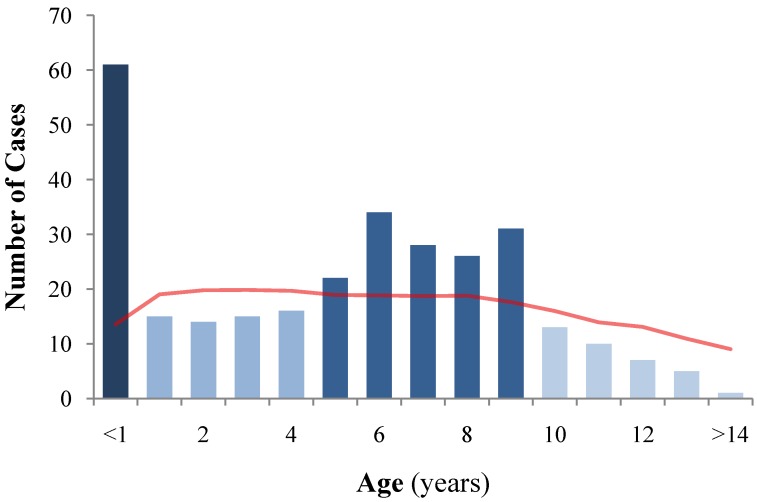
Age distribution of 298 dogs diagnosed with leptospirosis in Switzerland.

Note: The number of cases is represented in blue bars, with the age classes characterized with different shades of blue. The red line depicts the age distribution of the whole Swiss canine population, based on the mandatory dog registration database (ANIS).

During the 10 years of the study, 298 dogs were diagnosed with leptospirosis at the Veterinary Teaching Hospital of the Vetsuisse Faculty University of Bern and they were included in this report. This corresponds to an overall annual incidence rate of 5.88 diagnosed cases of leptospirosis per 100,000 dogs per year. Median age of the affected dogs was 6.3 years (1.8–8.7), the youngest dog being 1.5 month old and the oldest dog 14.4 years old. Age distribution is represented in Figure 1, indicating 61 puppies (<1 year, 20.5%), 60 young adults (1–4 years, 20.1%), 141 middle-aged dogs (5–9 years, 47.3%), and 36 older dogs (>10 years, 12.1%). Compared to the normal dog population of the ANIS dog registration database, puppies were markedly over-represented and older dogs under-represented (*P* < 0.001). 

Gender distribution indicated a clear over-representation of males (68.1%) compared to females (31.9%), in contrast to the whole canine hospital population consisting of 51.9% males and 48.1% females (*P* < 0.001) or to the ANIS dog registration database with 49.2% males and 50.8% females (*P* < 0.001). Neutering status was similar to the hospital population for males (31.0% of affected males were neutered, compared to 31.4% in the male hospital population; (*P* = 0.97)) but sexually intact females were markedly under-represented (28.4% in affected females compared to 47.4% in the female hospital population; *P* < 0.001). 

Two hundred and fifty-seven dogs (86.2%) were pure-bred dogs from 91 different breeds and 41 dogs (13.8%) were mixed-breed dogs. Most dogs were from large breeds with a median body weight of 20.4 kg (11.0–30.0 kg). 

#### 3.1.2. Disease: Diagnosis, Organ Manifestations, and Outcome

**Figure 2:**
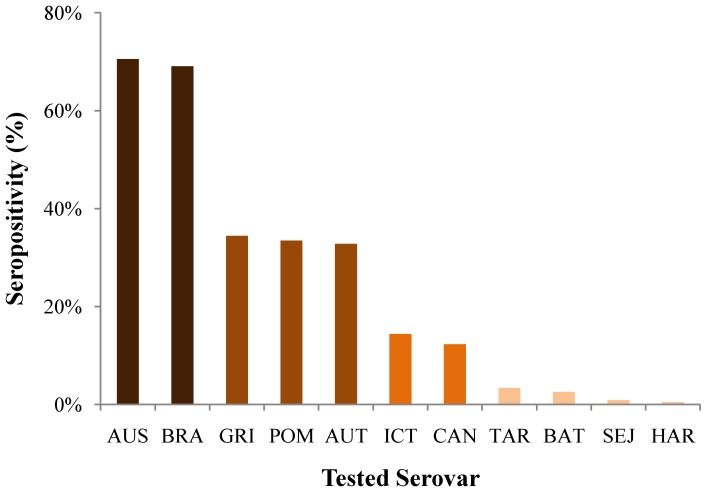
MAT seropositivity for 11 serovars tested in 259 dogs diagnosed with leptospirosis in Switzerland.

Notes: The results include grouped data obtained from paired sample serology (% seroconversion) in 132 dogs and single sample serology (% dogs with titer ≥1:800) in 127 dogs, the pattern being similar for both groups of dogs. The serology panel included following serovars: *L. interrogans* serovars Australis (AUS), Bratislava (BRA), Autumnalis (AUT), Pomona (POM), Icterohaemorrhagiae (ICT), Canicola (CAN), Tarassovi (TAR), Bataviae (BAT), Sejroe (SEJ), Hardjo (HAR), and *L. kirschneri* serovar Grippotyphosa (GRI).

In 132 dogs (44.3%) the clinical suspicion of leptospirosis was confirmed with paired MAT serology, in 127 dogs (42.6%) with single MAT serology, in six dogs (2.0%) with IgM ELISA, in 11 dogs (3.7%) with PCR, and in 22 dogs (7.4%) with histopathology. The rates of seropositivity to the 11 individual serovars tested are reported in Figure 2. Of the 259 dogs confirmed by MAT serology, 70.5% and 69.1% tested positive to the two main serovars Australis and Bratislava, respectively, both serovars belonging to the same serogroup Australis. An intermediate group with the serovars Grippotyphosa, Pomona, and Autumnalis tested positive in 33%–34% of the dogs. 

Vaccination history was available for 251 dogs (84.2%). Of these, 239 (95.2%) had been vaccinated against leptospirosis using a standard bivalent vaccine containing the serovars Canicola and Icterohaemorrhagiae in the past, and 220 (87.6%) within the last 12 months, according to the current recommendations for canine vaccinations. Seropositivity to these these vaccine serovars was only seen in 14.4% (Icterohaemorrhagiae) and 12.3% (Canicola) of the tested dogs, with the highest titer to one of these two serovars observed in only three dogs not vaccinated for more than 12 months.

Seventy-three dogs (24.5%) were affected by only one of the four main organ manifestations (renal, hepatic, pulmonary, or hemorrhagic), 130 dogs (43.6%) with two, 69 dogs (23.2%) with three, and 26 dogs (8.7%) had all four organ systems affected. Except for one dog diagnosed with isolated liver manifestation, all dogs diagnosed with leptospirosis showed evidence of renal involvement (297/298, 99.7%), with a median serum creatinine concentration of 686 µmol/L (462–977) (normal range, 53–120 µmol/L). At presentation, 65 dogs (22.4%) had mild AKI, 132 dogs (45.5%) moderate AKI, and 93 dogs (32.1%) severe AKI, with oligo-anuria present in 95 dogs (31.9%). Hepatic involvement was seen in 104 dogs (35.4%) and it was severe in 71 of them (24.1%). Pulmonary manifestation was diagnosed in 203 dogs (68.8%) and DIC in 40 dogs (18.4%). 

Out of the 298 dogs diagnosed with leptospirosis, 169 (56.7%) survived to discharge, 30 (10.1%) died, and 99 (33.2%) were euthanized for medical and/or financial reasons.

In 2007, introduction of a standardized diagnostic workup of new therapeutic options changed the perceived profile of the disease and its outcome. Organ involvement and odds ratios for survival are therefore described for the 256 dogs diagnosed since that time (2007–2012) and summarized in Table 1. 

**Table 1:** Main organ system manifestations in 256 dogs diagnosed with leptospirosis between 2007 and 2012 and univariate odds ratios (OR) for negative outcome (death or euthanasia).

Organ Involvement	N affected/N total	% affected	OR for negative outcome	95% CI	P
Renal	255/256	99.6%	n/a	n/a	n/a
Serum creatinine at presentation			1.0019	1.0011–1.0027	<0.001
Pulmonary	194/253	76.7%	3.6	1.8–7.2	<0.001
Hepatic	66/254	26.0%	16.3	7.7–34.5	<0.001
Hemorrhagic	38/209	18.2%	7.9	3.4–18.4	<0.001

Note: The odds ratio for negative outcome was not calculated for renal involvement since almost all dogs were affected. However, the effect of the degree of renal failure on outcome was evaluated with the OR for the serum creatinine concentration that was positively associated with death.

#### 3.1.3. Geographic Distribution of the Cases 

**Figure 3:**
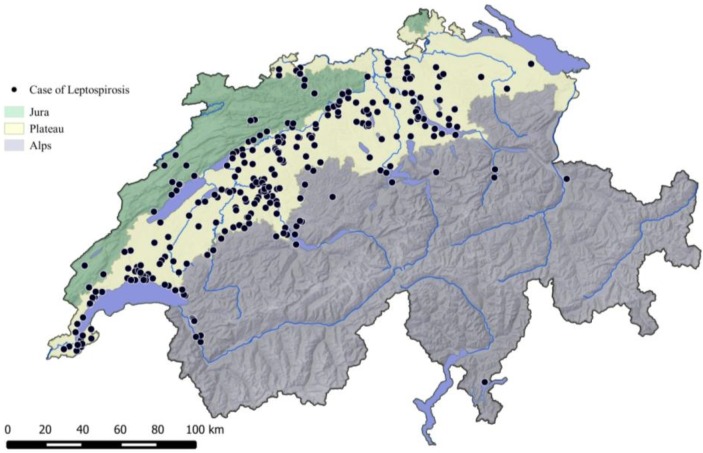
Geographic representation of the origin of 298 dogs diagnosed with leptospirosis (2003–2012) and their localization in the three main bioregions of Switzerland.

Notes: The surface area of Switzerland was divided in Jura (north-west), Plateau (centre), and Alps (south), according to the bioregion definition of the Swiss Federal Office for the Environment. The distribution of the cases diagnosed with canine leptospirosis and the distribution of the corresponding human population was as follows: Jura (11% of the canine cases, 23% of the human population), Plateau (75% of the canine cases, 66% of the human population), Alps (14% of the canine cases, 11% of the human population).

**Figure 4:**
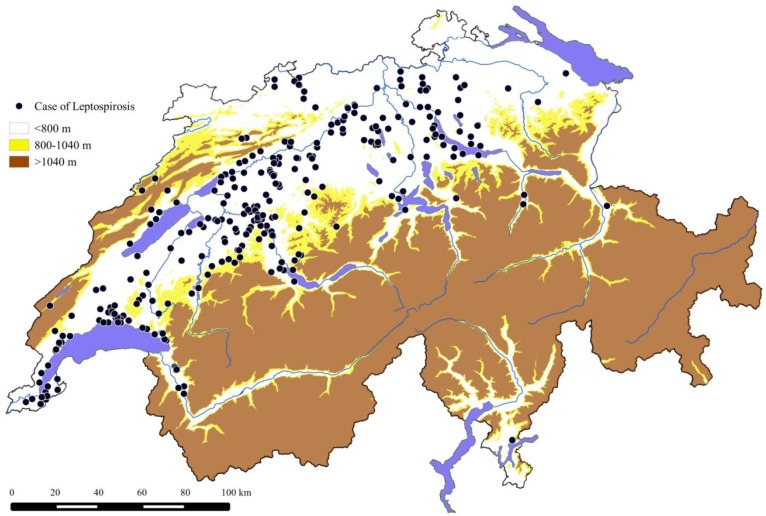
Geographic representation of the origin of 298 dogs diagnosed with leptospirosis (2003–2012) and their relationship to the altitude.

Notes: Following altitude zones are represented in this figure: white: <800 m (including 95% of the cases); yellow: 800–1040 m (95th–99th percentiles of the cases); and brown: >1040 m (1% of the cases).

The majority of the cases diagnosed with leptospirosis during the 10 years covered by this report were living in lower parts of the country as represented in Figure 3 and Figure 4. Two hundred and nineteen dogs (75.5%) were originating from the bioregion Plateau (27% of the national surface area, 66% of the human population); 31 dogs (10.7%) from the Jura (10% of the national surface area, 23% of the human population); and 40 dogs (13.8%) from the Alps (63% of the national surface area, 11% of the human population) (Figure 4). The median altitude of the animals location was 485 m (431–567) above sea level, with 95% of the dogs coming from altitudes <800 m (Figure 4). 

Two hundred and seventy-eight of 298 cases (93.3%) were originating from 10 main cantons (BE, VD, ZH, AG, FR, GE, SO, BL, NE, LU), from which >5 cases had been diagnosed during the 10 years of the study. The average annual incidence of diagnosis in these main cantons was 6.2 cases/100,000 dogs/year (5.1–8.4). The highest peak incidence rate measured in the group of the main cantons was 28.1 diagnosed cases/100,000 dogs/year for canton Aargau (AG) in 2010. The cantonal distribution of the dogs diagnosed with leptospirosis is presented in Table 2. The distribution of the canine and human population and the geoclimatic characteristics possibly associated with disease occurrence are also presented for the 26 cantons. Altitude and yearly rainfall were lower and average temperature higher for the most affected cantons than for the others. 

#### 3.1.4. Incidence and Trend over the Years

**Figure 5:**
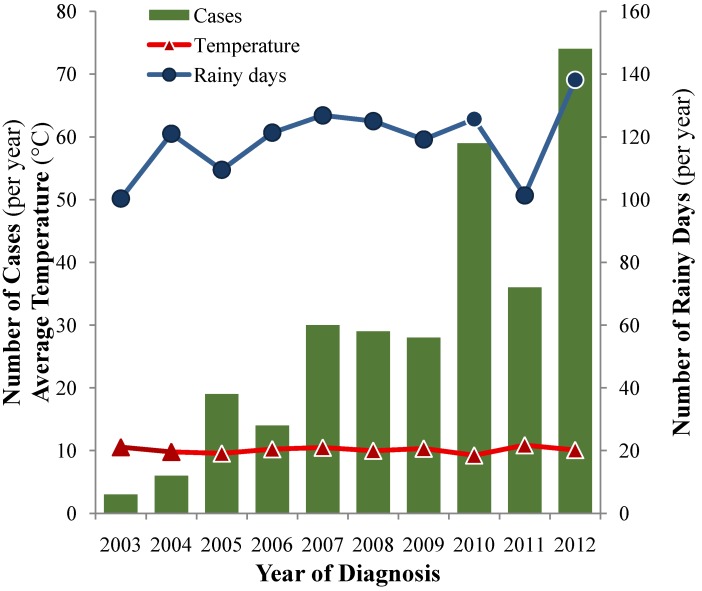
Annual number of cases of canine leptospirosis diagnosed at the University of Bern from 2003–2012 and association with country-wide average annual temperature and number of rainy days.

The number of dogs diagnosed with leptospirosis at the University of Bern has been markedly increasing over the 10 years of the study (Figure 5), with the highest number of cases recorded in 2012 (74/298, 24.8%), corresponding to a peak annual incidence rate of 14.3 diagnosed cases of leptospirosis/100,000 dogs/year for all of Switzerland. The proportionate morbidity ratio increased similarly from 0.96 (2003) to 23.0 (2012) cases per 1000 dogs presented to the teaching hospital for veterinary care.

**Table 2:** Cantonal distribution of 298 cases of canine leptospirosis and relationship to geoclimatic data.

Canton	Cases	Annual Incidence of Diagnosis		Canine Population	Human Population	Altitude	Temperature	Rainfall
	*n*	Average	Peak	*n* × 1000	*n* × 1000	Average (m)	°C	mm/year
		*n*/100,000 dogs/y						
Most affected cantons (>5 cases/10y)								
BE	90	13.6	27.1	66.4	963	1198	6.1	115.6
VD	59	9.8	21.7	60.0	672	827	8.5	100.9
ZH	32	5.5	17.3	57.7	1308	533	9.5	97.5
AG	25	6.4	28.1	39.1	582	476	9.7	88.3
FR	19	8.8	18.6	21.5	263	856	8.2	96.9
GE	14	4.9	13.9	28.7	438	419	10.9	73.9
SO	13	6.1	14.0	21.4	250	630	9.1	95.8
BL	9	5.0	22.0	18.1	269	521	9.7	87.3
NE	9	7.3	24.4	12.3	170	919	7.8	106.7
LU	8	3.9	24.4	20.5	364	777	8.3	113.7
Median (IQR)		6.2	21.9	25.1	401	704	8.8	97.2
		(5.1–8.4)	(17.6–24.4)	(20.7–53.1)	(265–649)	(524–849)	(8.2–9.6)	(90.2–105.3)
Least affected cantons (<5 cases/10y)								
GL	2	7.9	79.0	2.6	38	1589	3.9	145.5
SG	2	0.7	3.7	27.1	466	1000	7.5	121.2
SZ	2	2.7	13.6	7.3	141	1082	6.6	147.8
TG	2	1.1	5.7	17.5	238	495	9.5	83.8
GR	1	0.7	7.5	13.4	189	2021	1.9	95.1
NW	1	6.2	61.5	1.6	40	1077	6.3	135.4
TI	1	0.4	3.8	26.4	329	1412	6.3	138.4
VS	1	0.5	4.5	22.2	299	2140	1.7	109.2
ZG	1	2.3	23.3	4.3	109	651	8.6	117.3
AI	0	0.0	0.0	0.9	16	1126	7.2	136.1
AR	0	0.0	0.0	4.5	53	935	7.8	126.0
BS	0	0.0	0.0	5.0	185	522	10.9	71.6
JU	0	0.0	0.0	8.3	70	690	8.7	101.3
OW	0	0.0	0.0	1.8	34	1329	5.4	141.0
SH	0	0.0	0.0	4.7	75	538	9.4	75.9
UR	0	0.0	0.0	1.6	35	1896	2.4	134.3
Median (IQR)		0.4	3.7	4.9	92	1080	6.9	123.6
		(0.0–1.4)	(0.0–9.0)	(2.4–14.4)	(40–201)	(680–1456)	(5.0–8.6)	(99.7–136.7)
P		<0.001	0.004	<0.001	<0.001	0.02	0.03	0.04

Correlations between numbers of cases diagnosed and average temperature or yearly rainfall were very weak at the country level, at the level of the 10 main cantons, or at the level of canton of Bern with *r*^2^ 0.001–0.060 (temperature) and 0.044–0.150 (rainfall). A moderate correlation with climatic data was obtained for the number of summer days (*r*^2^ 0.245, *P* < 0.001) or the number of rainy days (*r*^2^ 0.376, *P* < 0.001). However, the numbers of winter days or freezing days and sun exposure did not seem to correlate with the annual case number.

#### 3.1.5. Seasonality and Climatic Factors

**Figure 6:**
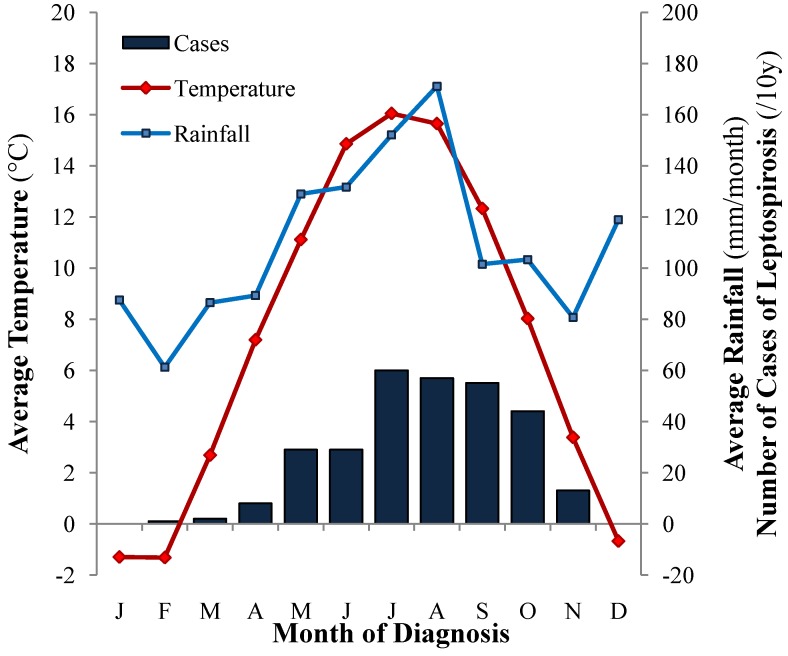
Seasonal distribution of 298 cases of canine leptospirosis in Switzerland and corresponding average monthly temperature and rainfall.

Most cases of canine leptospirosis (274/298, 91.9%) were diagnosed during the summer–fall period (May–October) as shown in Figure 6. Over the 10 years of the study, only three cases (1.0%) were diagnosed in the first quarter of the year, as opposed to 66 (22.1%) in the second, 172 (57.7%) in the third, and 57 (19.1%) in the fourth quarter of the year. The months with higher incidences of leptospirosis were also the months with the highest average temperature and rainfall (Figure 6). Monthly case numbers correlated strongly with the average monthly temperature (*r*^2^ 0.73, *P* < 0.001) and moderately with the average rainfall (*r*^2^ 0.39, *P* < 0.001).

**Figure 7:**
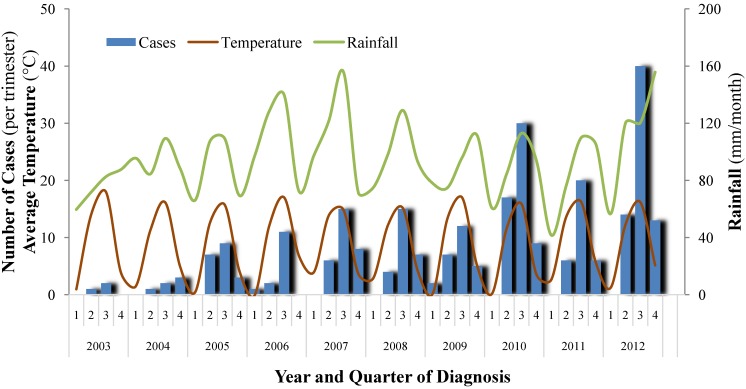
Distribution of 256 cases of leptospirosis by quarters for the 10 mainly affected cantons (2003–2012) and corresponding temperature and rainfall curves.

A detailed time-frame of the case distribution by quarter is shown in Figure 7, together with temperature and rainfall. A moderate correlation can be observed between quarterly case numbers and temperature (*r*^2^ 0.37, *P* < 0.001) and between quarterly case numbers and rainfall (*r*^2^ 0.30, *P* < 0.001) for the 10 main cantons. Similar results were obtained when analyzing these data for the whole country or when restricting the data to the canton Bern only, in order to minimize recruitment bias. 

### 3.2. Discussion 

This study presents an overview on 298 cases of clinical canine leptospirosis in Switzerland over a 10-year period. The results indicate a significantly increasing incidence during that time. With 74 cases, the peak year 2012 stands in striking contrast to an earlier study conducted at the same institution, reporting 11 cases during the five-year period 1992–1996 [35]. These data support the hypothesis that canine leptospirosis is a re-emerging disease in this area of temperate climate.

Describing cases from a single institution, the present study does not pretend to represent uniformly the whole Swiss canine population. Furthermore, not all dogs with leptospirosis are seen by a veterinarian or referred to a specialist for advanced care and increasing awareness for the disease may have biased the yearly incidence rates of diagnosed cases. However, since yearly case numbers and correlations with geoclimatic parameters yielded similar results when restricted to the subpopulations of the most represented cantons or solely to the canton of the authors’ institution, the authors feel confident that a possible geographic recruitment bias remained minimal and did not affect the main findings of the study. Additionally, the diagnostic effort and the inclusion criteria defined in the previous study [35] were very similar to the ones used for this report, indicating a true increase of the incidence of canine leptospirosis in Switzerland. 

The seasonal pattern of infections with predominance of the summer and autumn months are in agreement with previous studies [36,37,38]. The correlations observed between temperature, rainfall, and monthly case numbers support a role for climatic conditions, likely facilitating survival and development of both leptospires and their rodent vectors in the environment. Changes in these climatic conditions (e.g., due to global warming) could therefore potentially lead to an increase in the yearly incidence of leptospirosis. However, environmental factors have not changed drastically over the past 10 years and correlations with yearly incidence rates were weak for most climatic parameters tested. The marked increase in the incidence of canine leptospirosis is therefore only partially to be explained with climatic variations. Other factors including changes in population size, activity, and habitat of various host species favoring survival and/or transmission of leptospires must therefore exist. In the rural areas of Berlin, a dramatically growing population of wild boars harboring leptospires has been described simultaneously with an increasing incidence of canine leptospirosis [30]. A similar observation was made in another area of Germany, when an unusually mild winter in 2006/2007 favored the growth of the vole population, leading to a resurgence of human leptospirosis [39].

Our results show that canine leptospirosis is especially prevalent in certain areas of Switzerland, with only a small number of cases from the mountain bioregions of the Jura and the Alps. In these bioregions, a harsher climate with lower temperatures year round hinders survival and/or proliferation of shed leptospires and influences the population of wildlife hosts. On the Plateau bioregion, infections seem to be concentrated around lakes and urban sites, although this densely built area does not really allow these two parameters to be analyzed more closely. The spread into urban areas has previously been reported in Europe and USA and it may be related to the proliferation of rodents as well as the urbanization of previously wild areas [39]. 

The dog is the natural reservoir for serovar Canicola but the occurrence of this serovar has declined in Europe since dogs are vaccinated routinely [40]. Most infections currently documented in dogs are from other (incidental) serovars, with differences between geographic areas [21]. European reports describe mostly the serovars Australis, Grippotyphosa, and occasionally Pomona [24]. During the whole duration of the study, only bivalent vaccines against serovars Canicola and Icterohaemorrhagiae have been available in Switzerland, providing therefore inadequate protection against the encountered serovars, as evidenced with the high rate of vaccine coverage in the affected dogs from the current study. However, high titers against serovars Australis and Pomona were already described 15 years ago in Switzerland at the authors’ institution, making a recent serovar shift the principal cause of increasing incidence of leptospirosis very unlikely [35]. Obviously shifts in infecting leptospires could well happen at the level of strains and not of serovars. The definition of serovars being somehow artificial anyway, its characterization with serological data alone is imprecise [23,33]. 

Clinical manifestations of canine leptospirosis are very similar to the human disease, including acute renal and hepatic failure, hemostatic disturbances, and an increase in pulmonary involvement over the last few years, closely resembling the severe pulmonary form of leptospirosis (SPFL) described in humans [23,31,41]. Severity of disease and organ involvement may vary, and current data do not support an association with the inciting serovar, although exact serovar identification based on culture and strain isolation have not been performed in these studies that were only based on serology results [36]. The high rate of SPFL found in the present study and in reports from other countries, may originate from a real shift of organ involvement or it could be at least partially due to increased awareness and diagnostic effort [30]. Rates of mortality from SPFL in human medicine are as high as 30%–70%, similar to our findings that indicate a clear association with negative outcome [42,43,44].

In the present study, intact male dogs are overrepresented, in accordance with previous publications [2]. The increased risk may be explained partly by increased outdoor activity and by canine male specific behavior including sniffing and licking of urine, potentially favoring dog-to-dog transmission. A similar gender predisposition has been described in humans, where it was initially thought to be associated with occupational and outdoor activities [45], although recent data suggest that other factors including hormonal influences must be involved as well [46]. Our results further indicate that puppies seem to be at increased risk of leptospirosis with 20.5% of dogs in this study being under the age of 1 year. This may be due to overexposure to the environment during the socialization process or to a temporal gap in immunity. A generalized immune compromise (e.g., secondary to malnutrition or poor development) was however not a feature of the dogs from this study, where most of the young dogs were very fit until a few days prior to the presentation for veterinary care (data not shown). 

The precise role of the dog in the epidemiology of leptospirosis is difficult to establish and remains controversial [19]. It is likely that marked differences exist between areas with different geographic, wildlife, social, and economic features, and that the role of the dog may vary depending on these parameters. A close contact of many humans with dogs and their full integration in the household bring the potential of high exposure, particularly in industrialized countries. Detection of leptospires by PCR in the urine of 1.5%–8% of clinically healthy dogs can justify a role as potential reservoir and vector for the canine species [12,14].

Increasing numbers of human cases of leptospirosis have been reported from non-tropical countries including Germany and Ireland [47], paralleling an increasing number of diseased dogs in Germany [48,49]. While transmission of infectious agents from the dog to humans is certainly possible, most of the evidence for the role of the dog as a vector of infection remains indirect and based on similarities in seroreactivity patterns [13,19]. 

Exposure of veterinary professionals has been evaluated in several studies indicating seroreactivity in 2.5% of veterinarians treating small, large, and exotic animals [50]. Another study performed at the authors’ institution could however not show any evidence of exposure in owners of sick dogs and in veterinary staff treating a high caseload of dogs with leptospirosis, indicating that direct transmission of leptospirosis from clinically affected dogs is uncommon when precautionary measures are taken and standard hygiene protocols are respected [18]. To the authors’ best knowledge, none of the human caretakers of the 298 clinically sick dogs from the present study were infected, despite repeated and sometimes very close physical contact. However, since uncomplicated and asymptomatic courses of disease cannot be excluded, the exact role of the dog as a zoonotic vector in Switzerland cannot be further defined based on these data.

On the other side, this report shows that dogs suffer from clinical manifestations similar to the ones described in humans and that canine leptospirosis is associated with a high mortality rate despite a high level of care. A role of model for the human disease may therefore be postulated and it may be useful for investigations into the pathogenesis and the therapy of uncommon or new manifestations such as the SPFL, for example [31,51]. 

Although not directly comparable, the high incidence rates of diagnosed cases described in the present study (median of peak cantonal incidence rate of 22 diagnosed cases/100,000 dogs/year) are closer to incidence rates described for humans in tropical regions or during epidemic outbreaks following natural disasters. The calculated incidence rates are certainly underestimating the true incidence and represent only the tip of the iceberg. Our hospital treats only approximately half of the severely affected dogs nationwide and the number of dogs not requiring referral to a specialized institution is even more difficult to estimate. 

The high incidence rates from the present study stand however in marked contrast with a low incidence in humans (0.05 cases/100,000 humans/year in Switzerland, 1988–1998) [15,52]; a non-existing problem in food animals under official control and mandatory reporting; and no obvious evidence of a severe problem in wild animal species. Newer data or trends in the annual incidence of human cases are unfortunately not available for Switzerland and the disease has been reported to be likely underdiagnosed in this country [52]. It is also likely that higher levels of disease are needed for it to become apparent clinically in some wildlife species. Other indicators of a possible latent problem have however been reported, including a high prevalence of renal carriers in rodents of the city of Zurich and the recent description of a few affected beavers deceased in the waters of the Plateau [53,54]. In this area, the dog seems therefore to be the only obvious evidence for the presence of leptospirosis and these canine data indicate that leptospires are more widespread than commonly thought in Europe. This report should thus increase awareness of this “neglected tropical disease” in non-tropical areas, and contribute to a more realistic and evidence-based risk estimation in all animal species and in humans. 

## 4. Conclusions 

The results of this study indicate that canine leptospirosis is a re-emerging disease in the temperate climate zone of Switzerland with increasing incidence over the past few years, independent of environmental factors. Canine leptospirosis has similar clinical manifestations and outcomes as in humans for which it may be a model. Next to being a potential vector of disease, the dog may be indicative of high infectious pressure from the environment. Increasing numbers of active infections in our canine population should therefore heighten the awareness of the disease for humans and other animal species and support early diagnostic efforts and therapeutic intervention.
